# cAMP Signaling Affects Irreversible Attachment During Biofilm Formation by *Pseudomonas aeruginosa* PAO1

**DOI:** 10.1264/jsme2.ME13151

**Published:** 2014-02-19

**Authors:** Kaori Ono, Rie Oka, Masanori Toyofuku, Ayane Sakaguchi, Masakaze Hamada, Shiomi Yoshida, Nobuhiko Nomura

**Affiliations:** 1 Graduate School of Life and Environmental Sciences, University of Tsukuba, 1–1–1 Tennodai, Tsukuba, Ibaraki 305–8572, Japan

**Keywords:** cyclic AMP, attachment, biofilm, *Pseudomonas aeruginosa*

## Abstract

*Pseudomonas aeruginosa* responds to environmental changes and regulates its life cycle from planktonic to biofilm modes of growth. The control of cell attachment to surfaces is one of the critical processes that determine this transition. Environmental signals are typically relayed to the cytoplasm by second messenger systems. We here demonstrated that the second messenger, cAMP, regulated the attachment of cells. Our results suggest cAMP inhibited the transition from reversible to irreversible attachment. Further analyses revealed that cell surface hydrophobicity, one of the key factors in cell attachment, was altered by cAMP.

Biofilm formation is regulated by physicochemical and biological signals (1, 7, 28). The first step of biofilm formation by *P. aeruginosa* is the attachment phase, in which cells reversibly attach to surfaces and eventually become irreversibly attached (19, 22). Bacteria subsequently produce extracellular polysaccharides such as pel, psl, and alginate, which support the formation of biofilms (9, 11). The transition from reversible to irreversible attachment is critical for biofilm formation (5); therefore, understanding the factors that control this process is important. The bacterial second messenger, c-di-GMP, was recently shown to control biofilm formation in response to environmental influences (6, 13, 25). cAMP has also been studied extensively as a typical second messenger, which is conserved among diverse organisms (2). Intracellular cAMP concentrations in *P. aeruginosa* are controlled by CyaA and CyaB adenylate cyclases or CpdA phosphodiesterase, which synthesize or degrade cAMP, respectively (29, 34). cAMP binds with the cAMP binding protein Vfr, and regulates more than 200 genes (21, 34). A recent study demonstrated that cAMP was involved in the dispersal of biofilms (16). However, the mechanisms by which cAMP affects biofilm formation remain poorly understood. We here showed that cAMP affected the first step of biofilm formation by inhibiting cell attachment to surfaces.

One of the difficulties in studying the effects of cAMP on biofilms is that the accumulation of cAMP requires specific conditions, such as a low calcium environment (24, 34). To overcome this limitation, we constructed an in-frame deletion mutant of the cAMP phosphodiesterase gene Δ*cpdA*, which constitutively accumulates cAMP, by a previously described protocol (31). The cAMP binding protein, Vfr, was also deleted to examine the involvement of the cAMP binding protein. pG19II plasmids carrying deletion cassettes were transferred into *P. aeruginosa* PAO1 (15) by conjugation using *Escherichia coli* S17-1 (20, 30). Gene deletion was confirmed by PCR as previously described (31). The strains and primers used in this study are listed in Tables S1 and S2, respectively. cAMP levels were measured using an immunoassay (cAMP-Screen^®^ 96-well Immunoassay System; Applied Biosystems) following a previously described protocol (10). Intracellular cAMP levels were compared by normalizing cAMP concentrations with total cell protein measured by the Bradford protein assay (3). As expected, intercellular cAMP levels were 5-fold higher in the Δ*cpdA* mutant than in the wild-type (WT) after a 12-h cultivation in Luria-Bertani (LB) medium at 37°C under shaking conditions at 200 rpm (stationary phase of growth) (data not shown). Intracellular cAMP levels were also similar between the *cpdA*-complemented strain (Δ*cpdA*/pBBR1MCS5-*cpdA*, Table S1 [17]) and the WT (data not shown).

To investigate the effect of cAMP accumulation on biofilm formation, WT and the mutants were inoculated in LB medium in 96-well microtiter plates (23), and statically cultivated at 37°C. Cell density was measured at an optical density of 600 nm (OD_600_), and biofilm formation was quantified using 0.1% crystal violet (23). Biofilm formation by the Δ*cpdA* mutants was significantly less than that by the WT, which indicated that cAMP inhibited biofilm formation (Fig. 1A). Similar growth curves were observed for all the strains tested in this study (Fig. 1B); therefore, the effect of *cpdA* on biofilm formation was not due to differences in growth. Biofilm formation recovered in the *cpdA*-complemented strain (Δ*cpdA*/pBBR1MCS5-*cpdA*). The effect of cAMP was not observed in the Δ*cpdA*Δ*vfr* double mutant, which indicated that cAMP inhibited biofilm formation through its receptor Vfr. The deletion of Vfr alone had no effect on biofilm formation (data not shown).

To further investigate the effect of cAMP accumulation on biofilm formation, the attachment phase was examined in more detail by measuring the cell populations of reversibly or irreversibly attached cells on surfaces (5). Reversible attachment is defined as a moving cell that interacts with a surface via the cell pole, while irreversible attachment is defined as a cell oriented parallel to the surface (14, 18, 19, 36). Previous studies demonstrated that reversible or irreversible attached cells were easily distinguishable under a microscope (4, 5) (Fig. S1). A culture solution at the stationary phase of growth, as shown in Fig. 1B, was inoculated in fresh LB medium to obtain 1:100 dilutions, and initial attachment was recorded for 30 s after a 1-h incubation at 37°C. Three fields were recorded for each strain in 3 independent experiments. The irreversible attachment of Δ*cpdA* was significantly less than that of the WT, while reversible attachment was similar among the strains (Fig. 2). This result suggests that the accumulation of cAMP inhibited irreversible attachment, and consequently decreased biofilm formation. Irreversible attachment was restored in Δ*cpdA*/pBBR1MCS5-*cpdA* and in the Δ*cpdA* Δ*vfr* mutant, which confirmed the involvement of cAMP signaling.

The physical properties of cells, such as cell hydrophobicity, are known to affect the attachment of cells to their substratum (12, 32, 33, 35). The microbial adhesion to hydrocarbons (MATH) test was adapted to investigate cell hydrophobicity (26, 27). After a 12-h culture in LB medium, as shown in Fig. 1B, cells were collected and washed in phosphate buffered saline (PBS) buffer twice, and were suspended in the same buffer at an OD_600_ of 0.6. Bacterial cell suspensions were mixed with n-hexadecane at a ratio of 1:1 (volume), and cultured for 30 min at 30°C. Hydrophobicity was calculated from the difference in OD_600_ before and after incubation (8). The results obtained showed that the Δ*cpdA* mutant was more hydrophilic than the WT. Consistent with other results, cell hydrophobicity was restored in the *cpdA* complementary strain and in the Δ*cpdA*Δ*vfr* mutant (Fig. 3). These results demonstrated that cAMP altered cell hydrophobicity.

Taken together, our results demonstrate that the accumulation of cAMP inhibited the attachment phase of biofilm formation. Hence, cAMP, as well as c-di-GMP, control biofilm formation as second messengers in *P. aeruginosa*. While no significant differences were observed in reversible attachment between the cAMP-accumulating Δ*cpdA* strain and the WT, irreversible attachment was significantly inhibited in the Δ*cpdA* mutant. Therefore, cAMP may be involved in the transition from reversible to irreversible attachment. In reversible attachment, the cells are attached loosely by their poles and can move (5, 14). In contrast, irreversible attachment is accomplished when the cell firmly attaches to the surface (19, 22). The alteration observed in cell hydrophobicity in the Δ*cpdA* mutant may have inhibited the ability of cells to attach to surface, thereby inhibiting the transition to irreversible attachment. The negative effect of cAMP on biofilm formation is consistent with a previous study that reported the involvement of cAMP in biofilm dispersal (16). Our results further demonstrate that cAMP is also involved in the attachment of cells to surfaces, and provide an insight into how cells respond to environmental influences and regulate their life cycle.

## Supplementary material



## Figures and Tables

**Fig. 1:**
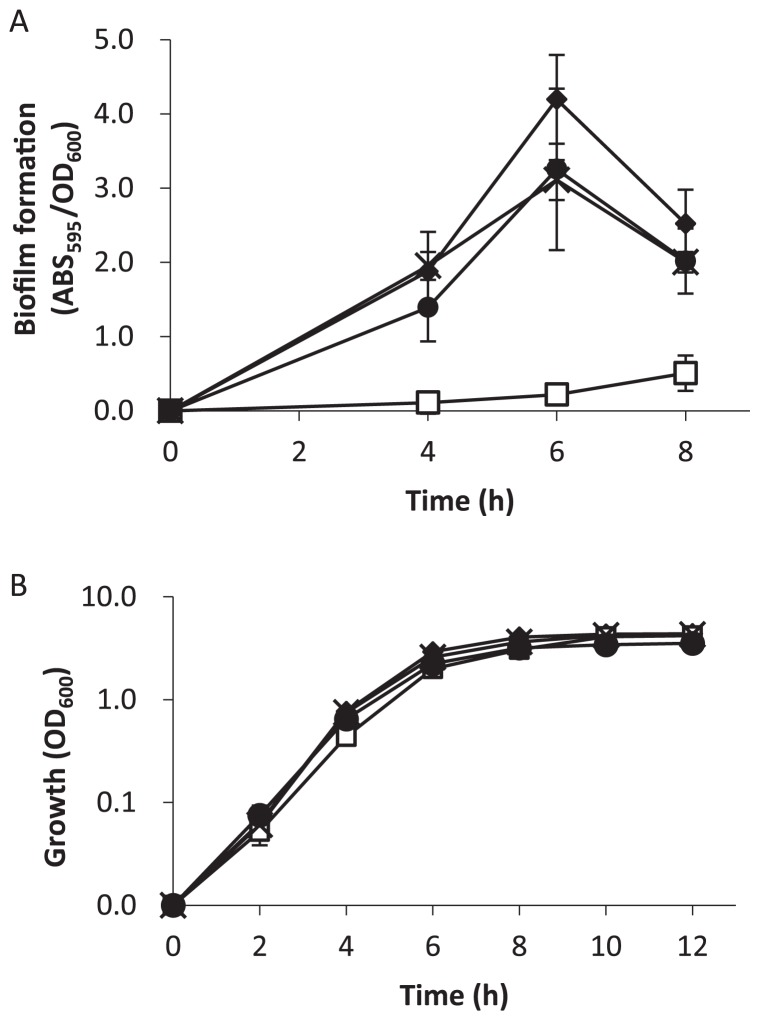
Biofilm formation (A) and growth curve (B) of *P. aeruginosa* PAO1 (solid diamond), Δ*cpdA* (open squires), Δ*cpdA*Δ*vfr* (solid circles), and Δ*cpdA*/pBBR1MCS5-*cpdA* (crosses). Biofilm formation (A) was evaluated by optical density (OD_600_) and crystal violet staining (ABS_595_) after cultivation in LB medium at 37°C under static conditions. A growth curve (B) was obtained during cultivation in LB medium at 37°C under shaking conditions at 200 rpm. Each value is the mean of three independent tests. Error bars indicate one standard deviation.

**Fig. 2:**
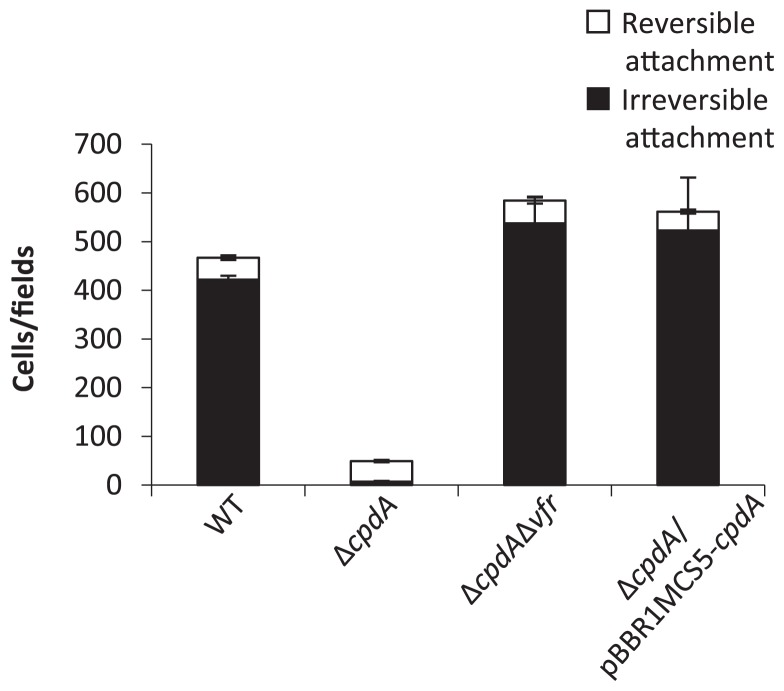
Quantification of the attachment to the polystyrene surface. The number of reversibly and irreversibly attached cells was microscopically counted for 30 s after a 1-h static culture at 37°C on a polystyrene plate. □ shows the amount of reversibly attached cells, and ■ shows the amount of irreversibly attached cells. Each value is the mean of three independent tests. Error bars indicate one standard deviation.

**Fig. 3:**
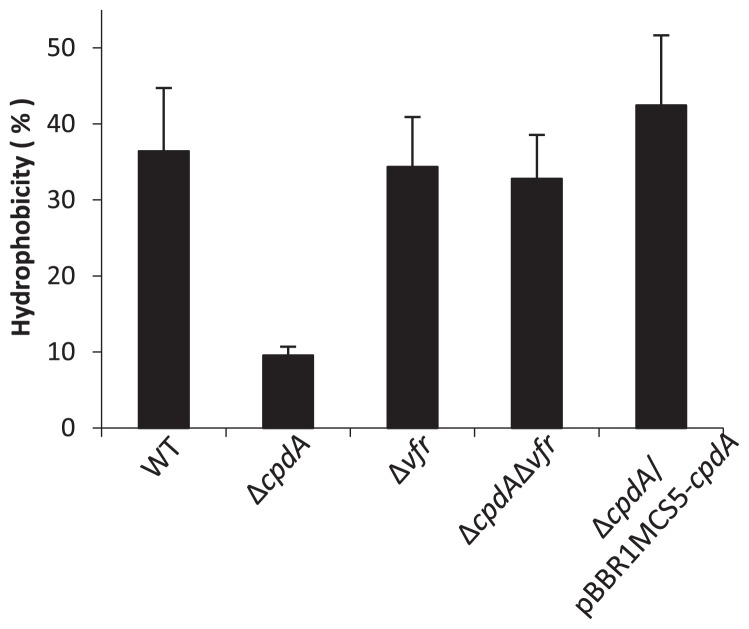
Hydrophobicity of *P. aeruginosa.* Hydrophobicity was measured using the MATH test, in which n-hexadecane was used as the organic solvent. Each value is the mean of three independent tests. Error bars indicate one standard deviation.
