# Supplementary material for: cAMP Signaling Affects Irreversible Attachment During Biofilm Formation by Pseudomonas aeruginosa PAO1
Source: Microbes Environ. 2014 Feb 19;29(1):104–6. doi: 10.1264/jsme2.ME13151 (PMC4041239; doi:10.1264/jsme2.ME13151)
Supplement: Supplementary file 1 [file 29_104_s1.pdf]

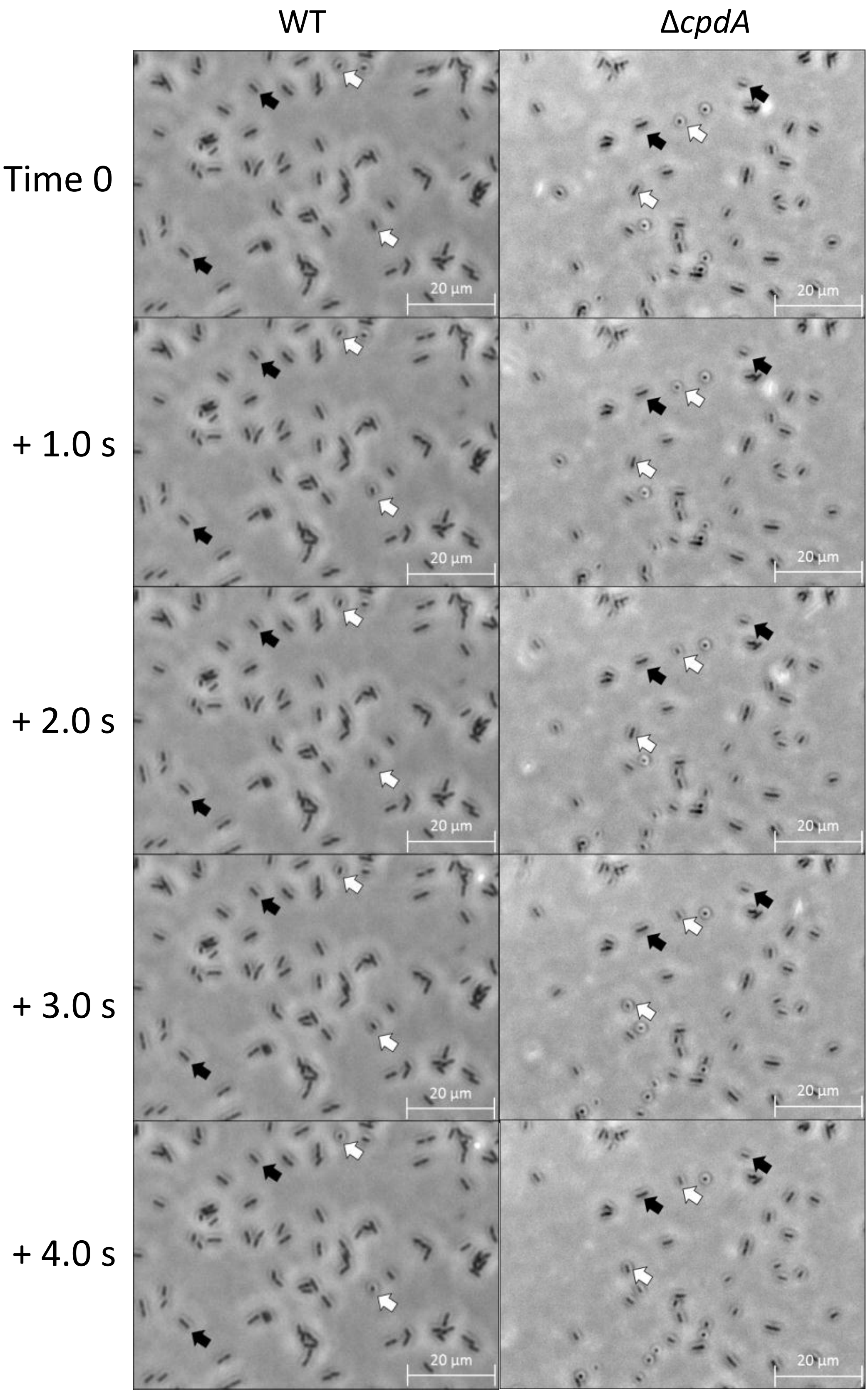

Fig.S1. Representative image of reversibly and irreversibly attached cells of *P. aeruginosa* WT and Δ*cpdA* to polystyrene surface. Phase-contrast images were recorded for 30 seconds after 1 h static culture on a polystyrene plate and the first 4 seconds are presented in this figure. White arrows indicate representative cells attached reversibly, and black arrows indicate representative cells attached irreversibly. Reversibly attached cells moves with one pole attached, while irreversibly attached cells are laid down on the surface and do not move.

Table S1 Strains and plasmids used in this study

Strains or plasmids	Relevant characteristics	Source or reference
strains		
P. aeruginosa		
PAO1	Wild type strain (WT)	1
Δ*cpdA*	PAO1 mutant with a deletion in the *cpdA* gene	This study
Δ*cpdA*Δ*vfr*	PAO1 mutant with a deletion in the *cpdA* and *vfr* genes	This study
Δ*cpdA*/pBBR1MCS5-*cpdA*	Δ*cpdA* complemented with pBBR1MCS5-*cpdA*	This study
E. coli		
JM109	*E. coli* strain for transformation	TaKaRa
S17-1	Mobilizer strain	4
plasmids		
pG19II	pK19mobsac derived suicide vector; *sacB* Gm^R	3
pG19IIΔ*cpdA*	*cpdA* deletion cassette in pG19II	This study
pG19IIΔ*vfr*	*vfr* deletion cassette in pG19II	This study
pBBR1MCS5	Broad-host-range cloning vector; Gm^R	2
pBBR1MCS5-*cpdA*	pBBR1MCS5 derivative carrying the *cpdA* gene	This study

References

1. Holloway, B. 1969. Genetics of *Pseudomonas*. Bacteriological reviews. 33:419–443.
2. Kovach, M.E., P.H. Elzer, D.S. Hill, G.T. Robertson, M.A. Farris, R.M. Roop, and K.M. Peterson. 1995. Four new derivatives of the broad-host-range cloning vector pBBR1MCS, carrying different antibiotic-resistance cassettes. Gene. 166:175–176.
3. Maseda, H., I. Sawada, K. Saito, H. Uchiyama, T. Nakae, and N. Nomura. 2004. Enhancement of the *mexAB-oprM* efflux pump expression by a quorumsensing autoinducer and its cancellation by a regulator, MexT, of the *mexEF-oprN* efflux pump operon in *Pseudomonas aeruginosa*. Antimicrob. Agents Chemother. 48:1320–1328.
4. Simon, R., M. O'Connell, M. Labes, and A. Pühler. 1986. Plasmid vector for the genetic analysis and manipulation of rhizobia and other gram-negative bacteria. Methods Enzymol. 118:640–659.

Table S2 Primers used in this study

Primers	Sequence*
Δ*cpdA*F1	5'-GGAATTCCTCGCCAACCCCTGGCTGC-3'
Δ*cpdA*R1	5'-GAAGATCTGAATGGCGTGACAAGGGGC-3'
Δ*cpdA*F2	5'-GAAGATCTCTTCGAAGTGGACTACGACACC-3'
Δ*cpdA*R2	5'-GCTCTAGAGCCATCGTCGATGACTTCCAGC-3'
Δ*vfr*F1	5'-GCGAAGCTTCATGCCCGTCAGATGCGAACGACGC-3'
Δ*vfr*R1	5'-GCAGATCAGCGGGTGCCGAAGACCACCATGG-3'
Δ*vfr*F2	5'-GCAGATCTACAGCACCCATGAAAAAGGCCGGTCGC-3'
Δ*vfr*R2	5'-GCGAAGCTTGAGAAGATGGACGAACTGGTGGCC-3'
*cpdA*F	5'-GGAATTCCAGGAGACGGCCCC-3'
*cpdA*R	5'-GCTCTAGAGCGCATGTCAGTATCCG-3'

*Restriction site are underlined.
